# Short-term inhalation of xenon during anesthesia for prevention of postoperative delirium in elderly patients undergoing laparoscopic radical colectomy: study protocol for a randomized controlled clinical trial

**DOI:** 10.1186/s13063-024-08290-8

**Published:** 2024-07-02

**Authors:** Yi Cheng, Ying Gao, Gu-Yue Liu, Fu‑Shan Xue, Mu Jin

**Affiliations:** 1 Department of Anesthesiology, Beijing Friendship Hospital, Capital Medical University, No. 95 Yongan Road, Xicheng District, Beijing, 100050 China; 2 Beijing Friendship Hospital, Capital Medical University, Beijing, 100050 China; 3 Department of Anesthesiology, Beijing Anzhen Hospital, Capital Medical University, No. 2 Anzhen Road, Chaoyang District, Beijing, 100029 China

**Keywords:** Xenon, Postoperative delirium, Laparoscopic radical colectomy, Elderly patients

## Abstract

**Background:**

Postoperative delirium (POD) is a common complication that is characterized by acute onset of impaired cognitive function and is associated with an increased mortality, a prolonged duration of hospital stay, and additional healthcare expenditures. The incidence of POD in elderly patients undergoing laparoscopic radical colectomy ranges from 8 to 54%. Xenon has been shown to provide neuroprotection in various neural injury models, but the clinical researches assessing the preventive effect of xenon inhalation on the occurrence of POD obtained controversial findings. This study aims to investigate the effects of a short xenon inhalation on the occurrence of POD in elderly patients undergoing laparoscopic radical colectomy.

**Methods/design:**

This is a prospective, randomized, controlled trial and 132 patients aged 65–80 years and scheduled for laparoscopic radical colectomy will be enrolled. The participants will be randomly assigned to either the control group or the xenon group (*n* = 66 in each group). The primary outcome will be the incidence of POD in the first 5 days after surgery. Secondary outcomes will include the subtype, severity, and duration of POD, postoperative pain score, Pittsburgh Sleep Quality Index (PQSI), perioperative non-delirium complications, and economic parameters. Additionally, the study will investigate the activation of microglial cells, expression of inflammatory factors in colon tissues, plasma inflammatory factors, and neurochemical markers.

**Discussion:**

Elderly patients undergoing laparoscopic radical colectomy are at a high risk of POD, with delayed postoperative recovery and increased healthcare costs. The primary objective of this study is to determine the preventive effect of a short xenon inhalation on the occurrence of POD in these patients.

**Trial registration:**

Chinese Clinical Trial Registry ChiCTR2300076666. Registered on October 16, 2023, http://www.chictr.org.cn.

**Supplementary Information:**

The online version contains supplementary material available at 10.1186/s13063-024-08290-8.

## Background

Colorectal cancer is the third most common cancer worldwide and is more prevalent among old population. More than 1.9 million new colorectal cancer cases and 935,000 deaths are estimated to occur in 2020, representing about one in 10 cancer cases and deaths [1]. Radical resection remains the primary treatment for colon cancer, and laparoscopic surgery has been shown to enhance recovery after surgery, reduce the occurrence of complications, and shorten the duration of hospital stay compared to open surgery [2, 3]. Both advances in minimally invasive surgical techniques and improvements in anesthetic care have provided more opportunities to undergo surgical treatment for elderly frail patients. However, available evidence indicates that elderly patients undergoing laparoscopic radical colectomy are at high risk of Postoperative delirium (POD), which is the main reason for delayed postoperative recovery and is associated with increased mortality and healthcare resource expenditure [4–8].

POD is a common and serious postoperative complication characterized by acute and fluctuating impairment in awareness and attention. Elderly patients with colon cancer are considered to be at a higher risk of POD due to the existence of various predisposing risk factors, such as preoperative cognitive impairment, malnutrition, comorbidities, polypharmacy, impaired functional status, and frailty, which accumulate and overlap with aging [7, 8]. In addition to the factors mentioned above, colectomy may trigger a cascade of events, including intestinal ischemia–reperfusion injury, disruption of the intestinal barrier, and inflammatory responses mediated through the inflammatory signaling in the gut-brain axis. This phenomenon is often referred to as the “two-hit model” of the surgical procedure [9]. These factors collectively exacerbate the onset of POD, especially in elderly patients who are more vulnerable [10]. Therefore, it is crucial to explore effective interventions of POD in these patients.

Updated guidelines issued by the European Society of Anesthesiology and American Geriatrics Society mainly focus on non-pharmacological interventions, such as preoperative physical conditioning and psychological interventions [11, 12], which may not be applicable for patients with colon cancer requiring surgical treatments as soon as possible. Thus, it seems more feasible to address the perioperative precipitating factors for these patients, especially in terms of clinically feasible pharmacological interventions.

Xenon, a noble gas with stable chemical properties and an excellent safety profile, has been used in clinical practice for over 70 years [13, 14]. It has been demonstrated that xenon can provide neuroprotection in vitro and in vivo neural injury models [15]. The neuroprotection of xenon is mainly attributable to antagonism at the N-methyl-D-aspartate (NMDA) subtype of the glutamate receptor [15]. Moreover, stable hemodynamic and rapid clearance properties of xenon make it less neurotoxic compared to conventionally used inhalational anesthetics [16–18]. In addition, xenon possesses an anti-inflammatory effect that may interfere with the pathogenesis of POD [19–21]. Considering that the neuroprotective mechanisms of xenon primarily target the pathophysiology of POD in elderly patients undergoing laparoscopic radical colectomy, we hypothesize that short-term xenon inhalation during anesthesia can reduce the occurrence of POD in these patients.

In addition to the incidence of POD, the outcome measures will also include several secondary endpoints, such as the subtype, severity, and duration of POD, postoperative pain score, Pittsburgh Sleep Quality Index(PSQI), perioperative non-delirium complications, and economic parameters. Furthermore, this study will measure the activation of microglial cells, expression of inflammatory factors in colon tissues, and levels of plasma inflammatory factors and neurochemical markers in order to explore the potential mechanisms of xenon to decrease the occurrence of POD.

## Methods/design

### Objectives and design

**Fig. 1:**
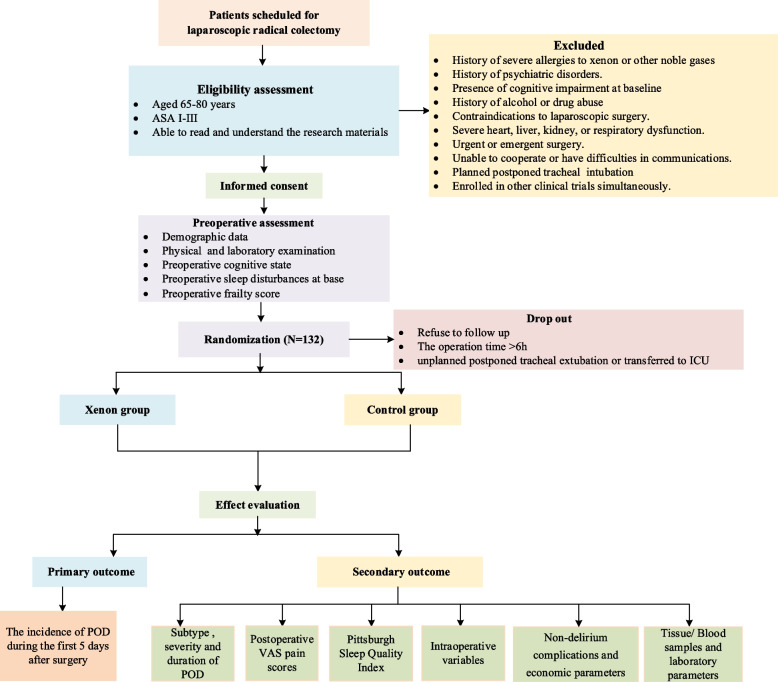
Flow diagram of the study

This prospective, randomized, controlled trial aims to test the hypothesis that short-term inhalation of xenon during anesthesia will reduce the incidence of POD in elderly patients undergoing elective laparoscopic radical colectomy. The study will enroll patients aged 65 to 80 years and scheduled for laparoscopic radical colectomy at Beijing Friendship Hospital, Capital Medical University, China. Participants will be randomly assigned to either the xenon group or the control group. The trial will adhere to the ethical principles and the trial protocol has been approved by the Ethics Committee of Beijing Friendship Hospital, Capital Medical University, China (Approval No: 2023-P2-100–03). The trial protocol has also been registered at the Chinese Clinical Trial Registry (Registration No: ChiCTR2300076666). Figure 1 shows the flow diagram of the study. The observatory will conduct screening based on established criteria and a pre-standard treatment plan. Data collection will commence with the accumulation of basic data and continue until the end of the follow-up (Table 1). We followed the SPIRIT reporting guidelines, and the (SPIRIT) 2013 Checklist is provided in Additional file 1.

**Table 1:**
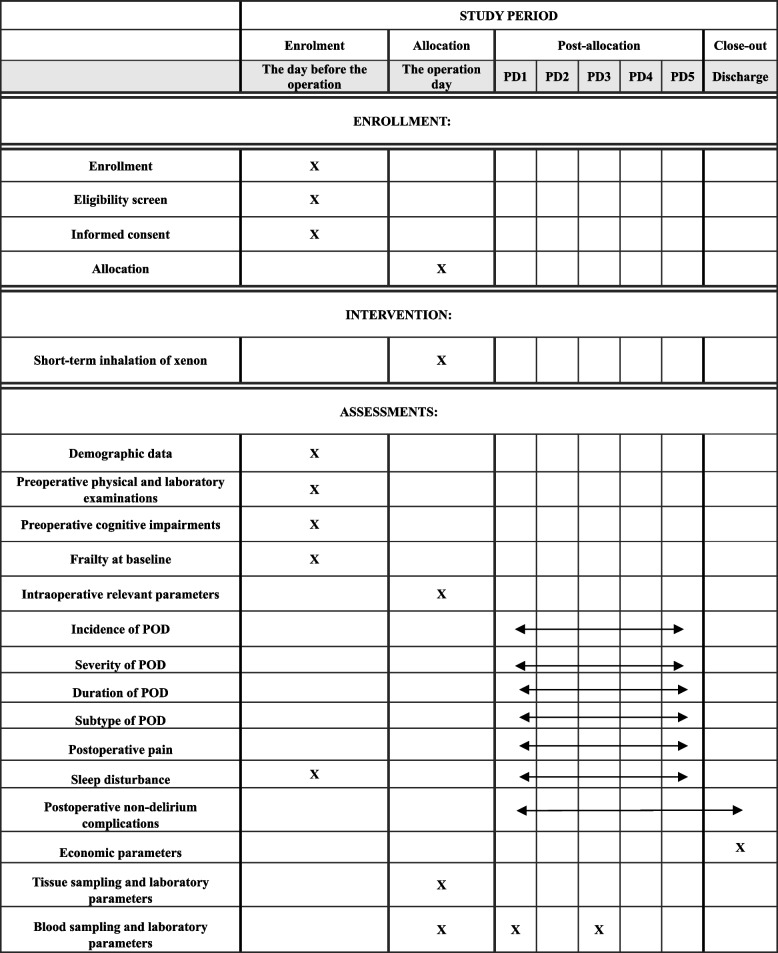
Standard Protocol Items: Recommendations for Interventional Trials (SPIRIT) schedule for enrollment, interventions, and assessments

*PD* Postoperative day

### Sample size calculations

The primary endpoint is the incidence of POD in the first 5 postoperative days. Based on the unpublished retrospective observational study enrolled 49 patients in Beijing Friendship Hospital, Capital Medical University, China, the incidence of POD in elderly patients undergoing elective laparoscopic radical colectomy was 35.7%, while it was 14.3% in the other elective laparoscopic surgery. We hypothesize that short-term inhalation of xenon during anesthesia can reduce the incidence of POD down to the same level as patients in the other elective laparoscopic surgery. To achieve a power of 80% with a two-tailed alpha of 0.05, 61 patients are at least needed in each group. Considering 8% potential dropouts or losses to follow-up, we will enroll 66 patients in each group, leading to a total sample size of 132 patients.

### Inclusion criteria

- Patients aged 65 to 80 years.
- Patients undergoing elective laparoscopic radical colectomy.
- American Society of Anesthesiologists (ASA) physical status classifications I to III.
- Able to read and understand the research materials.

### Exclusion criteria

- History of severe allergies to xenon or other anesthetics.
- History of psychiatric disorders, such as depression, anxiety disorder, schizophrenia, and mood disorders.
- Presence of cognitive impairment at baseline as screened with the Mini-Mental State Examination (MMSE) (< 24 for education less than postsecondary education, < 23 for below secondary education; < 20 for less than primary education, or < 18 for illiteracy) [22].
- History of alcohol or drug abuse.
- Contraindications to laparoscopic surgery.
- Severe heart, liver, kidney, or respiratory dysfunctions.
- Urgent or emergent surgery.
- Unable to cooperate or difficulties in communication.
- Planned postponed tracheal extubation or transferred to ICU after surgery.
- The operation time > 6 h.
- Enrolled in other clinical trials simultaneously.

### Recruitment

According to inclusion/exclusion criteria, patients undergoing elective laparoscopic radical colectomy in Beijing Friendship Hospital, Capital Medical University, China, will be screened by the anesthesiologist the day before surgery. The anesthesiologist will explain the process, risks and benefits of this trial, and the collection of participant data to the candidate patients who achieve the eligibility criteria. After consent is obtained, the patients will be recruited for this trial and sign the written informed consent by themselves or their authorized clients. The subject may withdraw from the study at any time and for any reason. Moreover, the trial should be terminated, if serious adverse events (AEs) occur. The reasons and circumstances for dropouts will be recorded in the Case Report Form (CRF).

Given that our hospital performs 400 to 500 elective laparoscopic radical colectomies per year, the trial period is set at 12 months to ensure the recruitment of a sufficient number of participants. The first participant was enrolled on November 20, 2023, and the enrollment period is expected to be completed by December 2024. When half of the cases have been collected, we will estimate the recruitment rate and adjust the trial period accordingly.

### Blinding and randomization

The eligible patients will be randomized to either the control group or the xenon group using a computer-generated randomization sequence. Randomization will be performed in a 1:1 ratio, and allocation will be concealed using sealed envelopes. Both the study subjects and investigators assessing outcomes will be blinded to group assignments, and the study statisticians will perform the data analysis after the trial is completed.

### Standard procedure

#### Preoperative evaluation

All patients will be visited and examined the day before surgery. Demographic data including education levels, medical history, current medications, and results of physical and laboratory examinations will be collected and analyzed. Signs and symptoms of preoperative cognitive impairments will be assessed preoperatively with MMSE [22]. Frailty of the patients at baseline will be measured by means of a multidimensional frailty score [23]. Preoperative sleep disturbances at base will be calculated with PSQI [24].

#### Interventions

After the sealed randomization envelopes are opened, patients will be randomly allocated to the two groups:

- Patients in the control group will receive conventional anesthesia management.
- Other than the conventional anesthesia management, patients in the xenon group will also receive 50% xenon inhalation in the first 15 after anesthesia induction, using a closed-circuit xenon delivery system integrated with the anesthesia machine, according to the methods described in our previous work [25].

#### Anesthesia management

Standard intraoperative monitoring will include continuous electrocardiogram, pulse oxygen saturation, bispectral index (BIS), end-tidal partial pressure of carbon dioxide (P_ET_CO_2_), and invasive blood pressure. Anesthesia will be induced with intravenous etomidate (0.1–0.4 mg/kg), sufentanil (0.3–0.5 μg/kg), and cisatracurium (0.1–0.2 mg/kg). After tracheal intubation, anesthesia will be maintained with continuous intravenous propofol and remifentanil, which are adjusted to maintain the BIS values between 40 and 60. The lungs will be mechanically ventilated with a FiO_2_ of 50% and positive end-expiratory pressure set at 5 cmH_2_O. P_ET_CO_2_ will be maintained between 35 and 45 mmHg by adjusting the tidal volume to 6–8 ml/kg and a respiratory rate of 10–15/min. In cases where hemodynamic stabilization (blood pressure change within 20% of baseline) cannot be achieved despite adequate volume loading, norepinephrine infusion will be administered. If the surgical procedure requires muscle relaxation, an additional 4 mg of cisatracurium will be intravenously administered. The administration of anesthetics will cease once all surgical interventions are completed. Extubation will be performed when spontaneous breathes is adequate and the patient responds to commands. Following extubation, patients will be transferred to the post-anesthesia care unit (PACU). Patients will be discharged from the PACU when their Steward scores reach > 4. Other perioperative care will be provided according to the current ERAS practices for colon surgery [3].

For the prevention of postoperative nausea and vomiting (PONV), dexamethasone 5 mg will be administered intravenously to both groups after anesthesia induction. At the end of the surgery, ondansetron 4 mg will also be administered intravenously to both groups. For patients with severe PONV (three or more episodes of vomits or inability to have activities of daily living [26]), postoperative rescue antiemetic therapy with additional ondansetron 4 mg will be intravenously administered.

For postoperative analgesia, all patients will receive wound infiltration with 0.5% ropivacaine and an intravenous infusion of 50 mg flurbiprofen axentil at the end of the operation. The goal of postoperative analgesic management is to keep a pain score of less than 4 on a 0–10 point visual analog scale (VAS). After the discharge from the operating room, the same mode of patient-controlled intravenous analgesia (PCIA) with sufentanil (1 μg/ml) will be provided for up to 2 days in both groups. The PCIA pump will be programmed to allow a bolus of 2 μg, 15-min lockout, and 1-h limit of four doses. As part of the postoperative multimodal analgesia regimen, 50 mg of flurbiprofen axetil every 24 h and 1 g of acetaminophen every 8 h were also administered intravenously. Additional rescue medication is 50 mg tramadol administered intravenously whenever the VAS pain score is more than 4 points.

### Study outcomes

#### Primary outcome: Incidence of POD as assessed by 3-Minute Diagnostic Interview for Confusion Assessment Method (3D-CAM)

The primary endpoint of this study is the incidence of POD during the first 5 days after surgery. The assessment will be conducted daily using a Chinese version of 3D-CAM by a blinded investigator during face-to-face follow-up. The Chinese version of 3D-CAM has been demonstrated to have high sensitivity (82.6–93.8%) and specificity (96.7–98.1%), along with strong reliability and validity [27]. A positive result on the Chinese version of 3D-CAM is based on the presence of four criteria: (1) acute onset and fluctuating course; (2) inattention; (3) altered levels of consciousness; and (4) disorganized thinking. POD is defined if both criteria 1 and 2 are present, with at least one of criteria 3 and 4.

#### Secondary outcomes

1. Severity of POD assessed with Delirium Rating Scale-Revised-98 (DRS-R-98).

The severity of POD will be assessed daily using the Chinese version of DRS-R-98. This scale is composed of 13 items, and the total score ranges from 0 to 39 points [28]. The severity of POD for each patient will be determined based on the highest value recorded on the scale during the first 5 postoperative days.

(2) Duration of POD assessed by 3D-CAM.

Duration of POD will be measured in terms of the number of days from the onset of delirium symptoms to the resolution of these symptoms.

(3) Subtype of POD assessed with the Richmond Agitation and Sedation Scale (RASS).

The subtype of POD will be defined based on the RASS score as follows [29]: hyperactive type, RASS score > 0; hypoactive type, RASS score < 0; mixed type, the hyperactive and hypoactive types occur alternately.

(4) Postoperative pain level during rest and physical activity measured with a 0–10 point VAS.

Postoperative pain level during rest and physical activity will be evaluated daily using the VAS [30]. Furthermore, the dosages of all opioids for postoperative pain control will be recorded and converted to morphine milligram equivalents.

(5) Sleep disturbance assessed with PQSI.

Patients with POD often experience pre- and postoperative sleep disturbances [31]. Therefore, PSQI scores will be determined at baseline and during the first 5 postoperative days. A sleep disorder will be defined if the PSQI score is higher than 5.

(6) Intraoperative relevant parameters.

Various intraoperative parameters, including blood loss, fluid volume, hypotension (defined as mean arterial pressure (MAP) below 65 mm Hg), hypertension [defined as systolic blood pressure (SBP) and/or diastolic blood pressure (DBP) greater than 30% of their baseline values, or SBP is ≥ 140 mmHg and/or (DBP) is ≥ 90 mmHg, tachycardia (defined as heart rate > 100 bpm), bradycardia (defined as heart rate < 50 bpm), arrhythmia and operation time will be recorded.

(7) Postoperative non-delirium complications and economic parameters.

The postoperative non-delirium complications, such as postoperative nausea and vomiting, pulmonary infection, myocardial infarction, and renal failure, will be documented. Economic parameters, including hospital stays, total hospital costs, and anesthesia-related expenditures, will also be recorded.

(8) Tissue/blood sampling and laboratory parameters.

Given the potential correlation between the inflammatory response and POD [32], the study will examine the activation of microglial cells, the levels of inflammatory cytokines including tumor necrosis factor (TNF-α) and interleukin-6 (IL-6) in the colon tissues for all patients. Additionally, the blood samples will be collected at baseline, after anesthetic induction, postoperative days 1 and 3, and will be analyzed for markers of glial injury including serum protein S100β, neuron-specific enolase (NSE), inflammatory cytokines (TNF-α and IL-6), and the indicators of intestinal permeability including serum D-lactic acid (D-LAC) and diamine oxidase (DAO).

### Data management

Data management in this study will adhere to the principles of Good Clinical Practice. The trained personnel will record all data into CRF using a double-entry procedure. The CRF will be promptly completed once the source document information is available and can be available on the clinical trial registration website of this trial (https://www.chictr.org.cn/showproj.html?proj=199575).

The Scientific Research Management Committee (SRMC) comprises an anesthesiologist, a scientific researcher, and a statistician. The SRMC’s primary responsibilities include data analysis and management, protocol improvement, and outcome adjudication. All data collection will be completed and secured by the anesthesiologist and the follow-up doctor. The data manager will meticulously review all recorded data, including demographic dates, inclusion criteria, exclusion criteria, dropout criteria, and any missing values. In the case of uncertainties or discrepancies, a “Data Query Form” will be generated and submitted to the quality control personnel. The researcher will provide written responses and fill the Data Query Form, which will then be returned to the data manager. It is essential to maintain the strictest confidentiality when handling the “Data Query Form.”

Instances not adhering to the protocol, such as failed operations, inability to complete the primary endpoint assessment, or deviations from standard procedures, will be retained. Upon completion of nearly half of the cases by each group, unblinding and interim analysis will be conducted by the SRMC statistician. If the interim analysis, post-unblinding, aligns with the hypothesis, the trial will proceed. In cases of inconsistency or contradiction, the expert committee will be consulted to determine whether to continue, terminate, or expand the sample size of the trial. Upon completion of the trial, the original data and results will be submitted to the SRMC, and electronic information will be securely entered into a password-protected mailbox. Public sharing of this information will be withheld until the results are published. All original records, including medical information, informed consent, CRF, and related files, will be stored and preserved for 10 years to enhance participant retention and ensure complete follow-up. Subsequently, these records will be destroyed in accordance with hospital standards.

### Statistical analysis

Statistical analysis will be conducted using SPSS 23.0 software by qualified statisticians from the clinical research center of Beijing Friendship Hospital. Due to the need for repeated evaluations of patients during the trial process, which may affect patient compliance, we will analyze the outcomes using both the Full Analysis Set (FAS) and the Per-Protocol Set (PPS) and compare the results from both analyses. The FAS will include all participants who are randomized according to the intention-to-treat principle, regardless of whether they strictly adhered to the trial protocol. Missing values in the FAS will be processed using the Last Observation Carried Forward (LOCF) method. The PPS will be defined as the population who strictly followed the trial protocol. This dual approach ensures a comprehensive assessment of xenon’s effectiveness, considering both generalizability and protocol adherence.

All statistical tests will be 2-sided, and a *p* value < 0.05 will be considered to indicate statistical significance. The choice of statistical tests will depend on the types and distributions of the parametric data.

- The primary outcome, incidence of POD within the first postoperative 5 days, will be analyzed using *χ*^*2*^ tests.
- Categorical secondary outcomes, including subtypes of POD; the rates of intraoperative hypotension, hypertension, tachycardia, bradycardia, and arrhythmia; postoperative sleep disorder; and non-delirium complications, will also be analyzed with the* χ*^*2*^ test.
- Parametric data will also be tested for homogeneity of variance by using the Levene median test. If the data follow a normal distribution and exhibit homogeneity of variance, they will be expressed as mean ± standard deviation and compared between groups using the *t*-test. When data do not meet the criteria for normal distribution or have uneven variances, they will be presented as median (interquartile range) and compared between groups using the Mann–Whitney *U* test.
- The within-group comparisons of data at multiple time points will be performed with the repeated-measures ANOVA.
- Time-to-event outcomes, such as the duration of POD and hospital stay, will be analyzed using the log-rank test.
- The logistic regression analysis will be utilized to examine the factors influencing the primary outcome between groups while controlling for covariates, such as preoperative frailty score, postoperative pain, and pre- and postoperative sleep disturbances.

## Discussion

This study is a single-center, prospective, randomized, placebo-controlled trial aimed at determining whether short-term inhalation of xenon during anesthesia can reduce the occurrence of POD in elderly patients undergoing laparoscopic radical colectomy.

Xenon is an ideal inhalational anesthetic, with stable performance, a low blood-gas partition coefficient for rapid uptake and elimination, minimal cardiovascular effects, and a lack of organ-specific toxicity [16, 17]. In contrast to the conventionally general anesthetic agents, xenon has exhibited favorable properties, including organ protection and hemodynamic stability [18], which are particularly beneficial for elderly and high-risk surgical patients. Additionally, it has been shown that xenon offers neuroprotection in vitro and in vivo models of various neural injuries by preserving cerebral flow metabolism, inhibiting the accumulation of β-amyloid, and reducing the neurotoxicity of anesthetics [16]. Furthermore, it has been demonstrated the interaction of xenon with the immune system that directly interferes with the pathogenesis of POD [19–21]. Based on these identified potential benefits, it is presumed that xenon may prevent or decrease the occurrence of POD. However, the clinical trials in this field provide conflicting results [33–36].

To mitigate these disparities, several improvements have been in the protocol. Firstly, the heterogeneity among study subjects is a critical factor contributing to inconsistent research conclusions. For instance, while a randomized clinical trial in patients undergoing elective off-pump coronary artery bypass graft surgery demonstrated a significant reduction in POD incidence with 50–60% xenon anesthesia [34], similar benefits were not observed in studies involving on-pump cardiac surgery [35] or hip fracture surgery [36]. Variations in patient age, surgical types, and comorbidities contribute to these discrepancies. In order to avoid these confounding factors, we focus on elderly patients undergoing laparoscopic radical colectomy, a high-risk and extremely vulnerable patient group for POD [6–9]. And patients with surgery durations exceeding 6 h and those planned for postoperative ICU admission are excluded to minimize variability in surgical procedures and reduce selection bias. Secondly, practical considerations during trial implementation have been carefully taken into account. For example, administering multiple questionnaires over five consecutive days postoperatively may lead to patient fatigue and non-compliance. Therefore, outcomes will be analyzed using both FAS and PPS, providing comprehensive and robust results to better understand xenon's potential protective effects. This approach enhances the reliability of the trial and aids in identifying and addressing potential biases and data variability. And the logistic regression analysis will be utilized to examine the high-risk factors influencing the primary outcome between groups, such as preoperative frailty score, postoperative pain, and pre- and postoperative sleep disturbances. Moreover, considering the high cost of xenon is a significant barrier to its clinical research and widespread adoption, xenon will be administered only for a short time, that is, 1 h after anesthesia induction, a period associated with a higher likelihood of hemodynamic instability [37–39].

Despite the inability to blind the anesthesiologist due to the use of a closed-circuit xenon delivery system integrated with the anesthesia machine, strict adherence to a standardized hemodynamic treatment protocol will ensure equivalent management in both groups and minimize bias. Additionally, both investigators and study statisticians assessing primary and secondary outcomes will be blinded to group assignments.

This study aims to validate the hypothesis that a short xenon inhalation during anesthesia will reduce the occurrence of POD in elderly patients undergoing laparoscopic radical colectomy. Positive results could guide anesthesiologists in selecting appropriate anesthesia for specific patient subgroups, potentially justifying the cost associated with xenon administration to benefit this high-risk population.

## Trial status

The first participant was enrolled on November 20, 2023, and the first version was developed on October 10, 2023. The enrollment period is expected to be completed in December 2024. An additional period of 3 months is allocated for data evaluation, statistical analysis, and publication of the results. The protocol presented above is the third version, which underwent protocol revisions, specifically in the redefinition of secondary outcome indicators. The updated protocol version has been communicated to the Institutional Review Board (IRB) and has obtained their approval. To date, 30 participants have been recruited, and the trial is actively ongoing.

### Supplementary Information

Additional file 1. SPIRIT checklist.

## Data Availability

After the study is completed and the results is published, the data will be open to the public through email to the research team.

## Abbreviations

POD: Postoperative delirium
PQSI: Pittsburgh Sleep Quality Index
ERAS: Enhanced Recovery After Surgery
NMDA: N-methyl-D-aspartate
ASA: American Society of Anesthesiologists
MMSE: Mini-Mental State Examination
CRF: Case Report Form
PONV: Postoperative nausea and vomiting
VAS: Visual analog scale
BIS: Bispectral index
P_ET_CO_2_: End-tidal partial pressure of carbon dioxide
PCIA: Patient-controlled intravenous analgesia
3D-CAM: 3-Minute Diagnostic Interview for Confusion Assessment Method
DRS-R-98: Delirium Rating Scale-Revised-98
RASS: Richmond Agitation and Sedation Scale
TNF-α: Tumor necrosis factor
IL-6: Interleukin-6
NSE: Neuron-specific enolase
D-LAC: Serum D-lactic acid
DAO: Diamine oxidase
PD: Postoperative day
FAS: Full analysis set
PPS: Per-protocol set
